# Paramedic perceptions of decision-making when managing mental health-related presentations: a qualitative study

**DOI:** 10.1186/s12911-024-02768-w

**Published:** 2024-11-19

**Authors:** Kate Emond, George Mnatzaganian, Michael Savic, Dan I. Lubman, Melanie Bish

**Affiliations:** 1 La Trobe Rural Health School, La Trobe University, P.O. Box 199, Bendigo, VIC 3552 Australia; 2 Turning Point, Eastern Health, Melbourne, 3120 Australia; 3 Monash Addiction Research Centre, Eastern Health Clinical School, Monash University, Melbourne, 3128 Australia

**Keywords:** Decision-making, Mental health, Paramedic, Qualitative research

## Abstract

**Background:**

Mental health presentations account for a considerable proportion of paramedic workload; however, the decision-making involved in managing these cases is poorly understood. This study aimed to explore how paramedics perceive their clinical decision-making when managing mental health presentations.

**Methods:**

A qualitative descriptive study design was employed. Overall, 73 paramedics participated in semi structured interviews, and data were analyzed from transcribed interviews in NVivo.

**Results:**

Four themes emerged that reflected participants’ perceptions: the assessment process, experience, the use of documents and standard procedures, and consultation with other healthcare providers. There were conflicting perceptions about the clinical decision-making process, with perception of role having a potential impact. The dual process theory of clinical decision-making, which includes both analytical and intuitive approaches, was evident in the decision-making process.

**Conclusion:**

Incorporating dual process theory into education and training, which highlights the strengths and weaknesses of analytical and intuitive decision-making, may reduce clinical errors made by cognitive bias. To further support clinical decision-making, additional education and training are warranted to promote critical thinking and clarify the scope of practice and roles when attending to mental health-related presentations.

**Supplementary Information:**

The online version contains supplementary material available at 10.1186/s12911-024-02768-w.

## Background

Australian studies indicate that between 10% and 20% of ambulance presentations are related to mental health [1, 2] reflecting the increasing demand on paramedics as they become the first point of contact for individuals experiencing mental health crises. In 2022–23, there were 287,500 mental health-related emergency department presentations, with over 52% of patients arriving via ambulance [3]. These statistics emphasize the expanding responsibilities of paramedics, particularly in areas with limited access to specialized mental health care. In recent decades, paramedicine has experienced changes characterized by an expanded scope of practice and a shift towards tertiary education [4]. Australia has extended paramedic response models from solely addressing medical emergencies and transport to include integrated community health service delivery [5], with the intent to alleviate demands on the healthcare system and reduce hospital admissions [6]. These roles include extended care paramedics and community paramedics, who provide treatment and referral for low- to medium-acuity cases in various community and clinical settings, emphasizing the management of individuals in their own environment [7].

The evolving role of paramedicine has led to changes in workforce preparation, transitioning from vocational training to tertiary education, enabling the discipline to establish itself as a profession. With this professionalism, individual clinicians gain greater autonomy, leading to increased decision-making demands [8]. However, there is a paucity of research on professionalism in paramedicine [9]. O’Meara articulated the need for the establishment of paramedic professionalism over a decade ago [10]. With the introduction of Australian paramedic registration in 2018 through the Australian Health Practitioner Regulation Agency, professional codes of conduct and professional capabilities were enforced as a part of the registration process. These capabilities required paramedics to ‘use clinical reasoning and problem-solving skills to determine clinical judgements and appropriate actions’ [11]. The evidence needed to demonstrate this focus on analytical decision-making.

The capabilities also require paramedics to engage in professional behavior that is empathetic and non-discriminatory, regardless of the individual’s mental state [11]. However, paramedics often feel uncertain or ambivalent about their role in responding to mental health presentations, with some displaying judgemental attitudes that can negatively impact on patients’ experiences of care [12, 13]. Stigmatized attitudes toward mental illness among paramedics can significantly impact their decision-making and, ultimately, the recovery of individuals experiencing mental health issues. Research suggests that paramedics’ perspectives on their scope of practice in mental health-related presentations are influenced by several factors, including educational and workplace cultures, organizational policies, and public stigma [13], Similar to the general population, paramedics may hold negative attitudes towards patients with mental health issues [14, 15], and studies indicate that these attitudes can result in frustration, and uncertainty about their role, particularly in cases where mental illness is perceived as less urgent or complex compared to physical health emergencies [16, 17]. Stigmatized attitudes towards mental health may lead paramedics to marginalize mental health presentations, influencing their clinical decision-making. This may result in a preference for transport-focused responses over more proactive mental health care, ultimately affecting the quality of care provided.

Little attention has been paid to how paramedics use clinical decision-making when attending mental health presentations, despite these being common presentations, and there is no empirical evidence focused on paramedic clinical decision-making [4]. A study by Shaban analyzed clinical practice guidelines, policies, and legislation to understand what informed or influenced clinical decision-making, and found that clinical decision-making was complex, with inconsistencies between mandated policy and actual practice [18]. The results indicated a gap in paramedics’ knowledge of mental health and a need for additional skills and training to support clinical decision-making in this area [18].

A further review of the literature identified that there is insufficient discipline-specific evidence related to paramedic clinical decision-making concerning mental health presentations [19]. Additional research supports this finding, emphasizing the limited understanding of the impact of mental health legislation on the clinical practice and decision-making of paramedics, warranting further investigation [20, 21].

Clinical decision-making has been described as an unseen skill that critically defines a clinician’s performance and the efficacy of the clinical environment [22, 23]. Research on clinical decision-making across health disciplines is vast and diverse [24], making a universal definition difficult to attain. This process underpins paramedic practice, including performing clinical procedures, administering medications, assessing patient acuity, making decisions about transporting patients to health facilities, undertaking environmental and risk assessments, and determining whether to involve additional service providers such as police [25].

Despite this, research investigating clinical decision-making in paramedicine is sparse compared to other health disciplines [23]. A systematic review on nurse practitioners aimed to develop a definition and framework for clinical decision-making, defining it as ‘a contextual, continuous, and evolving process, where data are gathered, interpreted, and evaluated in order to select an evidence-based choice of action’ [26]. Although previous studies have identified the need to focus research on clinical decision-making in paramedic practice [24], there has been little agreement on how to define clinical decision-making in paramedicine, and more importantly, on how it is applied in professional practice. It has been suggested that naturalistic decision-making aligns itself well with paramedicine [8].

Naturalistic decision-making is congruent in emergency settings, characterized by prompt decisions influenced by situational variables, time pressure, limited resources, and competing priorities [8, 27]. However, its limitation lies in the lack of analytical thinking applied to decision-making. It has been reported that paramedic practice often precludes the opportunity to use an analytical approach due to these situational variables, stress, and time pressure [8].

Previous work has emphasized that although prompt decision-making has its benefits in emergency settings, it can only be achieved by experts, leading to reservations among novice clinicians [22]. Results from earlier studies demonstrate a strong and consistent association between clinical decision-making and dual process theory [28, 29]. Dual process theory recognizes two types of decision-making: intuitive and analytical [30]. The intuitive approach is heuristic, concrete, and highly automatic, requiring minimal cognitive effort: it is also highly context-driven and vulnerable to errors and bias [30, 31]. In contrast, the analytical approach applies a normative reasoning style, is abstract, requires considerable cognitive effort, and is less susceptible to errors and bias [30, 31].

The significance of this theory lies in its inclusion of both types of decision-making, with health professionals in emergency settings utilizing both approaches [30]. It also articulates the strengths and weaknesses of each approach, providing a learning opportunity for health professionals such as paramedics, to gain insight into the characteristics and vulnerabilities of these decision-making types [30]. Findings from a recent review support dual process theory as a model for clinical decision-making in paramedicine [28].

Identifying a model to support clinical decision-making in paramedicine is an important first step, as it provides opportunities to embed this model in education and training. This study represents Phase 2 of a mixed-methods study design. Phase 1 aimed to examine the confidence and preparedness of paramedics in Australia to manage mental health-related presentations [32]. To provide context for the quantitative findings of Phase 1 and illustrate the phenomenon under study, this study aimed to explore how paramedics perceived their clinical decision-making in managing mental health presentations, contributing to the literature in this important area of paramedic practice.

## Methods

This qualitative study explored the management of mental health presentations by paramedics.

### Participants

Phase 1 was a cross sectional study utilizing an online survey [32]. Permission was obtained from six of the eight states and territories in Australia (New South Wales, Northern Territory, Queensland, South Australia, Tasmania, and Victoria) ambulance services. Recruitment occurred in metropolitan, regional and rural areas. Numerous approaches were employed to recruit paramedics, including emails, e-bulletins and e-newsletters sent by their ambulance service, Paramedics Australasia’s newsletter and website, and paramedic social media. Upon completion of the survey in Phase 1, the number of participants (*n* = 1,230) provided an opportunity to recruit from this sample, and participants were invited to take part in an interview. Non-probability sampling was adopted for this study, specifically using incidental sampling. This involves the selection of participants based on their availability rather than predetermined criteria [33]. Those willing to have an interview provided their telephone contact details, which were stored in a separate database to their survey. Approximately 6% of survey participants agreed to be interviewed, resulting in 73 participants. A follow-up telephone call was conducted by the research assistant to each participant, explaining the interview process. The research assistant was employed by the research institution with a background in public health. Participants’ privacy and confidentiality were protected throughout the study. Personally identifiable information was replaced with unique identifiers. Access to the data was restricted to authorized researchers through password-protected files. Participants were provided with an information sheet at the start of the interview, outlining the study’s purpose, participation details, risks and benefits, withdrawal options, data handling, and confidentiality measures. Verbal consent was obtained at the beginning of interview to ensure participants’ agreement and understanding of the study procedures.

### Study design

This study was conducted utilizing a qualitative descriptive study design. This is a widely adopted design in health care research where a study aims to explore participants’ experiences and factors related to certain phenomena and is a useful approach where there is limited understanding of the phenomena being studied [34]. Qualitative descriptive study design is a flexible approach that enables a rich description of experiences and perceptions using language from the collected data [35]. It allows a detailed and rich understanding of phenomena that is sensitive to the complexity and diversity of experiences [36]. Authenticity of qualitative descriptive study design involves the ability to capture participants’ perceptions, and accurately analyze and represent the data [36]. This design aligns well with the collection of data through semi-structured interviews [37]. The Standards for Reporting Qualitative Research (SRQR) 21-item checklist for qualitative studies [38] was applied for this study to ensure clear and complete reporting of study conduct.

### Data collection

Individual interviews were conducted, using an in-depth semi-structured interview schedule. The interview schedule topics (Table 1) were developed from the validated and reliable survey data and literature review and was agreed upon by the research team (see supplementary materials). The questions focused on paramedics’ experiences with managing mental health related presentations and what guided their clinical decision-making.

**Table 1:** Semi-structured interview schedule topics

Interview schedule topic
How would you describe your level of knowledge and confidence in managing mental health related presentations?
Describe your level of preparedness in managing mental health related presentations
In relation to your pre-qualification education how much theory and training was received in mental health? How adequate was this theory and training to equip you to manage mental health related presentations?
What, if any, theory based, and clinical practice “on-road” training have you received about assessing and managing mental health related presentations?
What, if any, professional development theory and training is available currently for you about assessing and managing mental health related presentations?
What, if any, clinical practice guidelines do your ambulance service have to support your work with mental health related presentations?
Aside from education and training, what, if any, access do you have to specialist staff (example: mental health practitioners) to help you manage mental health related presentations?
Overall, is there anything else you think of that guides how you manage mental health related presentations?

Interviews were audio-recorded and undertaken by telephone, and, apart from the interviewer and participant, no-one else was present. The use of telephones for interviewing was selected as the participants were geographically dispersed and it allowed for a consistent approach for data collection. Each interview lasted an average of 56 min (range 27 to 79 min). The interviewers included the primary author and two research assistants, all of whom had no previous contact with the participants.

### Data analysis

Data were analyzed from transcribed interviews managed in NVivo [39] utilising the dualistic approach of inductive and deductive analyses. This approach was selected as the findings from the survey data in Phase 1 could be explored in more detail. Inductive analysis allowed codes to emerge developing into themes and findings to help make sense of the data [40]. Deductive analysis applied predetermined codes to the data from the literature review and survey data relating to the research aims [40]. This guided the development of an analytical codebook where codes were labelled, described, and defined with inclusions and exclusions identified. As there were three researchers analyzing the data, testing of the codebook was conducted. Testing and re-testing of the data, using the codebook as a guide, was completed until no new codes emerged, signifying a valid representation of the data. This process of code creation and testing ensured rigor in the analysis [41]. Coding identified themes in participant responses, and similar themes were linked. Through sorting, merging, deleting and reorganizing codes, the final themes emerged from the data.

### Human research ethics

Five human research ethics committees in three states and territories gave ethical approval to undertake the study; Eastern Health Human Research Ethics Committee Victoria (E122/0809), Monash University Human Research Ethics Committee Victoria, South Eastern Sydney Local Health District Human Research Ethics Committee New South Wales, Flinders University Social and Behavioural Research Ethics Committee South Australia South Australia Department for Health and Wellbeing Human Research Ethics Committee while three other states/territories accepted ethics approval from another state. Informed consent to participate was obtained prior to the commencement of the interviews, and was audio-recorded via telephone, as approved by the ethics committees.

## Results

The interviews captured a broad range of experiences around paramedics’ clinical decision-making when managing mental health-related presentations. In total, 73 paramedics with a mean age of 43.9 years (standard deviation = 10.56; ranging from 23 to 63) participated in the study. Approximately 43% worked in metropolitan cities, 23% in regional areas and almost 33% from rural and remote areas. Participants’ demographics and workplace information are presented in Table 2.

**Table 2:** Paramedic participants’ sociodemographic and workplace information

	*N* = 73	%
**Sex**		
Male	42	64.6
Female	23	35.4
**State/Territory of work**		
New South Wales	22	30.1
Northern Territory	8	11.0
Queensland	11	15.1
South Australia	5	6.8
Tasmania	8	11.0
Victoria	19	26.0
**Geographic location of work**		
Metropolitan	32	43.8
Regional	17	23.3
Rural	20	27.4
Remote	4	5.5
**Highest professional educational qualification**		
Non-university vocational training certificate	1	1.4
Diploma	20	27.4
Degree	32	43.8
Graduate Certificate	6	8.2
Graduate Diploma	13	17.8
Master’s Degree	1	1.4
**Operational role**		
Paramedic Manager	2	2.74
Paramedic	50	68.49
Intensive Care Paramedic	14	19.18
Extended Care Paramedic	6	8.22
Flight Paramedic	1	1.37

**Fig. 1:**
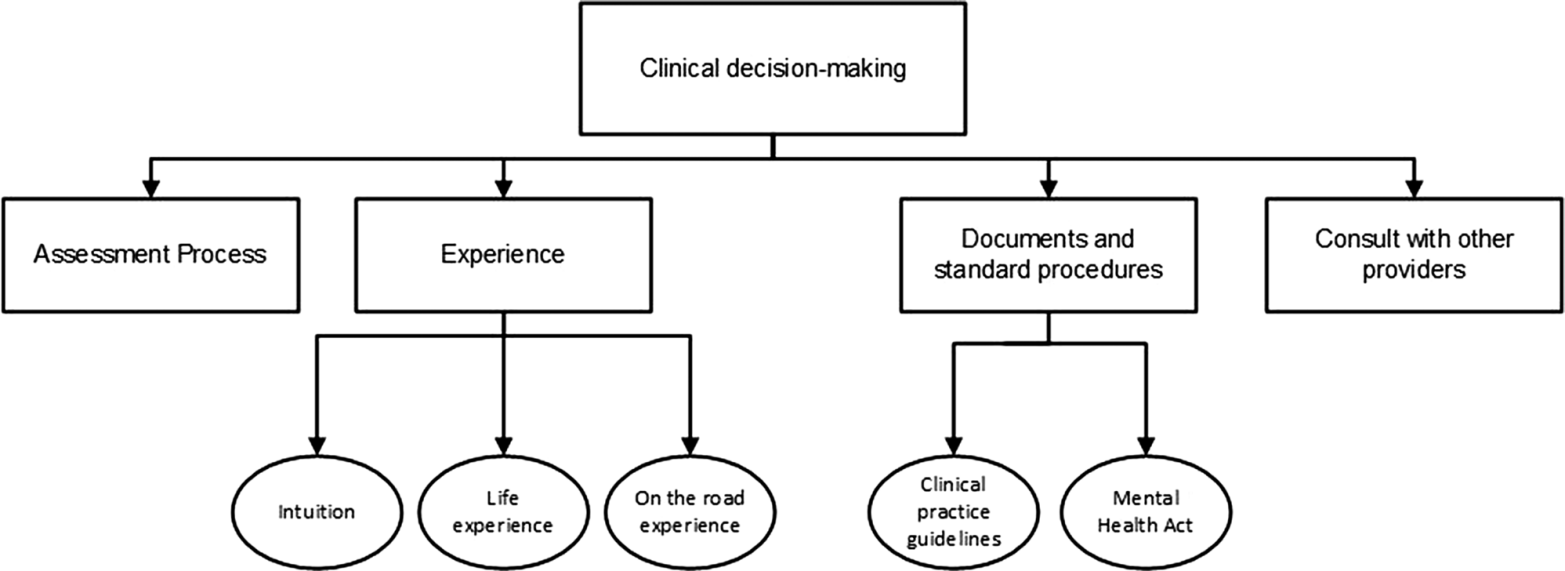
Themes and subthemes on clinical decision making

A total of 1582 first round codes were generated that were further developed resulting in the classification of 98 codes. Upon completion of the analysis, four interconnected themes were extracted from the data that reflect the participants’ experiences of clinical decision-making in managing mental health presentations: (i) the assessment process, (ii) experience, (iii) documents and standard procedures, and (iv) consultation with other providers. Sub-themes were identified from two of the themes as outlined in Fig. 1.

### Assessment process

The first theme illustrated paramedics’ experiences of clinical decision-making during the assessment process. The analysis revealed that listening to the patient and establishing their narrative featured prominently as influencing clinical decision-making: “tell us your story, tell us what’s happening so that we can get to the cause and understand what help we need to get you” (P 16). “The approach you take is it could well be non-judgmental. Everyone has got a story” (P 40). “Well, I basically listen. Quite often people want to be heard, and I’ll try and work with that” (P 4).

In contrast it was reported that participants did what the patient wanted despite the clinical presentation, as one participant stressed, “if they want to go to hospital, take them to hospital” (P 10).

Observation of patient body language was reported as influencing decision-making, although there were differing responses from participants which indicated body language was used as part of the overall assessment process “I’m also respectful of [unclear], reading body language” (P 73), and body language was used to make decisions without further analysis of the patient’s presentation, as participants declared “you’re just treating what’s in your face. There’s no time for analysis” (P 33), and “reading the body language and knowing when you’ve pushed the wrong buttons and that you’re going to have to, now, either leave or change, if you start reading that we’re getting nowhere, leave” (P 8).

Knowing a patient’s past history was seen as an important part of the assessment process and influenced clinical decision-making: “If they are known to have mental illness and then are drug and alcohol affected, I’d sort of be more likely to take them to hospital” (P 10). “Like obviously there’s certain regulars or individuals you learn how to handle over time. So that makes it easier. So, it’s just like dealing with your own family you see them that much” (P 12).

Health risk for patients were identified as influencing clinical decision-making, as one participant described “really acute situations where it’s life threatening, like suicide attempts, drug overdoses, take them to hospital so that they get help in that immediate situation because it’s life threatening” (P 7). The health risks towards a patient were also described in context of alcohol use; “the depressive symptoms come with being intoxicated, there’s not really much we can do other than take them to hospital and protect them from the symptoms associated with intoxication and obstruction of their airways and to vomiting” (P 13).

### Experience

A recurrent theme from the data analysis was a sense that an individual paramedic’s level of experience had a considerable influence on clinical decision-making. Three subthemes were identified as relating to the construct of experience: intuition, life experience, and ‘on the road’ experience. A prominent response was the use of intuition to guide clinical decision-making. This was described as a gut feeling, articulated as “generally, it’s gut feel for me as to how I deal with it” (P 9), with emphasis placed on its importance as reported, “the greatest thing I’ve said is trust your gut” (P 26).

Life experience was identified as influencing decision-making when there was personal experience of mental illness, as reported “My personal history, I had family members with mental conditions” (P 10). Being able to communicate with people was considered a skill that developed through life experience as opposed to a skill taught well: “the first skill is being able to talk to people. It’s something that is I don’t think taught very well, because it is a hard thing to teach, life experience comes with that” (P 20).

‘On the road’ experience referred to being exposed to mental health related presentations in the clinical setting, and clinical decision-making was influenced by this: ‘It’s just based on hundreds, hundreds if not thousands of experiences over the years of mental health patients’ (P 1). The outcomes of the decisions made based on these experiences were not described as beneficial or otherwise.

### Documents and standard procedures

There were two distinct categories in documents and standard procedures: clinical practice guidelines (CPGs), and the Mental Health Act (MHA). Contrasting views about the usefulness of CPGs to guide clinical decision-making were identified with CPGs providing set responses, and CPGs not being useful to guide clinical decision-making. Participants emphasized that CPGs provided protocols that determined set responses for some mental health related presentations. Set responses were identified in CPGs for sedation and restraint of agitated patients, as one participant described, “That’s just around the issue of restraints and sedation as to when we can and can’t do things” (P 24), and for suicidal patients: “It’s just working basically from protocols” (P 20). There were no set responses for mental health-related presentations that didn’t relate to suicide or agitation, however, participants did identify CPGs relating to mental health, in this case CPGs were viewed as not useful, particularly in the context of little education or training to support application of the guidelines: “Unless you’ve had teaching in the mental status assessment, reading that one page doesn’t mean anything really” (P 18), “they said learn them, I haven’t even looked at it. We need training” (P 11), “in the absence of a good training foundation, they’re pretty useless” (P 27).

It was identified that there was lack of clarity with some participants on which CPGs related to mental health presentations to support clinical decision-making. Talking about this issue, participants said: “I think we have protocols - I don’t know” (P 32); “I don’t think there is - from memory, there is nothing else. I may be wrong on that, but I don’t remember there being a specific guideline for mental health patients” (P 66).

The MHA was recognized as an important factor and influence in clinical decision-making, however, there was variation in the interpretation of the act, which indicated a potential misunderstanding of how legislation should be applied. There was a sense that the MHA applied to all mental health related presentations: “The only real indications of what we were supposed to do, how we were supposed to treat these people, is actually regulated by law, not by any clinical guidance” (P 20).

The MHA was used to transport people to hospital for assessment by another health professional: “Even though he came in voluntarily, I still put in an order. So that gives them four hours to assess him because that’s all it is for us, four hours. It gives us four hours window of opportunity from the moment they arrive at the hospital to assessment. So, yeah, involuntary treatment (P 11), and “A patient refuses to be transported to the hospital, that needs to be under the Mental Health Act (P 18). These clinical decisions were made in the context of paramedics perceiving their primary role as that of transport. Once transported it was identified the person would be assessed by another health professional. This perception of using the MHA for transport was further identified: “Many paramedics will say to a patient look, we can do this the hard way or the easy way; you either come with me voluntarily, or I’ll make you. But that’s coercion. That’s threatening that patient. I understand that I can’t do that” (P 33). While others had a distinct understanding of mental health legislation: “They do have rights, a patient does have a right to refuse - if they show us that they have capacity and they’re competent, there’s really no - we can’t take their rights away from them” (P 23).

### Consult with healthcare providers

Consultation with healthcare providers was established as informing and influencing clinical decision-making. Service providers included mental health services, emergency departments (ED), general practitioners (GP), and ambulance clinical consultants.

There were some conflicting views about consulting with other providers. One participant commented: “we don’t really have that ability to access any external providers, local GPs. Local GPs generally just want us to transport…we don’t really have any other alternative” (P 46). A possible explanation for this was expressed by other participants that reported: “I think also paramedics don’t know about what the services are that are trying to support these people” (P 32); and “We’re working in a bit of a vacuum. it’d be nice to know what you’re actually dealing with so you could actually go oh okay, that’s such and such’ (P 8). Other participants expressed belief that they didn’t need to consult with other providers and viewed their role as that of transport. As one participant said: “No we don’t need to, like I said, it’s a transport issue and getting some of the people if we can manage them and if they need restraint, obviously police are involved” (P 72).

In contrast consulting with other providers was seen as influencing clinical decision-making: “the mental health triage team, they can access the files or the history for that patient if they’ve got a history. So, you sort of make an informed decision” (P 10); and “Sometimes I will call the ED to see if they know the patient and if there is any information they can give (P 7)”. There was a sense that paramedics were still establishing the clinical supports they have in the broader healthcare system: “I think we’re only learning now to get our head around consulting with ED doctors and nurses” (P 32); and in relation to mental health services; “Yeah. Yeah, it’s okay in the instances where we use it. We just don’t utilise it a lot, yeah, so unfortunately” (P 5).

Few responses indicated the use of clinical consultants employed by ambulances services to inform clinical decision-making: “I can call up the clinical consultant, which as a doctor, I guess a consultant,24 hours a day. We can consult for stuff that may fall slightly outside of our guidelines” (P 4).

## Discussion

Four themes were identified in the data, describing participants’ perceptions of clinical decision-making when managing mental health presentations and the factors that influenced the clinical decision-making process.

The first theme associated clinical decision-making with the assessment process. The assessment process is a systematic collection and analysis of health-related information of an individual that encompasses clinical skills, observation, listening, interpreting and clinical judgement [42]. This finding aligns with previous work that defines the assessment process as a key factor in paramedic practice and effective clinical decision-making [43]. The analytical approach to clinical decision-making applied in the assessment process is defined as being highly reliable and less prone to errors [30].

Dual process theory recognizes analytical clinical decision-making alongside intuitive clinical decision-making. Findings from a recent review support dual process theory as a model for clinical decision-making in paramedicine [28]. Although participants did not articulate the use of dual process theory to support their clinical decision-making, properties of this are evident in their responses with both analytical and intuitive approaches adopted when attending to mental health related presentations. Recognition of different thinking approaches may reduce errors arising from cognitive bias. Bias in clinical practice is reported to negatively impact healthcare quality and clinical outcomes [44].

The second theme identified experience as an influence in clinical decision-making with three subthemes: intuition, life experience and ‘on the road’ experience. These findings support the use of both analytical and intuitive methods of clinical decision-making and are consistent with previous studies examining clinical decision-making amongst paramedics [28, 45]. The impact of previous experience on clinical decision-making has been observed in earlier studies which emphasized learnings developed through direct experiences [28, 45]. An important finding from the study was participants’ views on life experience in the decision-making process. Previous work on paramedic clinical decision-making in mental health articulated the importance of life experience and how that influences perceptions of reality [46]. Participants described managing mental health-related presentations based on their personal experiences, with differing perceptions and judgements of mental health and their role in attending to these presentations. This result may be partly explained by cognitive bias, which can occur when relying on life experience to make clinical judgements [47]. Another explanation for this differing view may be insufficient education and training about managing mental health related presentations and ambulance organisational cultures that emphasize an acute medical focus [4, 48]. Public stigma towards people with mental health related issues may also influence paramedics’ perception when making clinical decisions in this context. Public stigma has harmful effects on people experiencing mental health related issues and education is important in stigma change (Corrigan et al., 2012).

The third theme identified documents and standard procedures as influencing clinical decision-making, specifically CPGs and MHA legislation. The participants had conflicting perceptions on the usefulness of CPGs. These results are similar to previous work that found inconsistencies in paramedics’ views on guidelines and legislation, affecting how policy and practice was mandated [18].

Possible explanations for this may be related to experience, as earlier studies have highlighted that rigid judgments and strongly enforced use of guidelines were reported among novice paramedics [28, 49]. It may be that CPGs that are less prescriptive are viewed as not useful when there are gaps in knowledge to support clinical decision-making. Such gaps in knowledge regarding the management of mental health-related presentations have been previously highlighted [4, 16], indicating a rationale for the differing perceptions on the usefulness of CPGs. It has been emphasized that CPGs should serve as guide for clinical decision-making and not replace clinical judgment [50]; however, in situations characterized by chaos and pressure, guidelines may assist in reducing errors [51]. This supports participants’ perceptions of CPGs related to the suicidal patient, restraint, and sedation as being the most useful.

Another possible explanation for the differing perceptions on the usefulness of CPGs to support clinical decision-making may stem from paramedicine transitioning from algorithmic thinking with protocols, to critical thinking supported by CPGs. A change from protocols to CPGs has embedded the expectation of a broader application of profession practice that has increased autonomy and demands in decision-making [8]. CPGs to guide clinical decision-making have been cited as beneficial for patient care due to flexibility in thinking as opposed to assessing a patient against the limitations of a protocol [8]. The terms protocol and CPG are used interchangeably in participant responses with no clear distinction between the two. Similarly, a previous study found that the terms appeared to be used interchangeably by paramedics [52]. It may be that this distinction is yet to be embedded in education and training, coupled with a workforce that has recently moved to being a registered profession with roots still embedded in vocational training. Participants reported the need for training and education to support the use of CPGs related to mental health presentations. This insight aligns with the current debate as to whether paramedic education has kept pace with changes to the profession’s scope of practice [32, 48].

Participants recognized MHA legislation as an important influence on clinical decision-making but there were variations in interpreting the act. Differences included using legislation to impose an assessment on a person that refused to be assessed, with no dialog of the person meeting the criteria to be assessed involuntary, to recognizing the act was there to ensure a person was treated in the least restrictive way, indicating further investigating was required into clinical decision-making and legislation. A previous study examining paramedicine and mental health legislation supports this finding and identified that further work is needed to explore clinical decision-making and mental health legislative responsibilities [21]. In addition, it has been suggested that inconsistencies exist between CPGs and legislation, which can impact clinical decision-making [18].

The fourth and final theme identified consultation with other providers as informing and influencing clinical decision-making. Participants reported consultations helped to make informed decisions, demonstrating an analytical approach to clinical decision-making, yet there were some conflicting views with reports of not knowing how to access consultations with other providers. A previous study supports this finding by identifying deficiencies in interagency policy and established rapport between services [53], however, these findings were limited to one jurisdiction.

A recurring factor of importance in the findings is paramedics’ perception of their role when attending to mental health-related presentations. Differing views on this role may account for the inconsistent and contrasting results. The implications of contrasting perspectives about scope of practice and role have been previously examined, with findings suggesting that they reflect educational and workplace cultures,

organizational policies, and stigma towards people with mental illness [13]. Addressing these areas to develop consistency in the perception of role has the potential to support consistency in clinical decision-making.

The findings of this study have important implications for paramedic practice, education, and future research. In practice, the identification of both analytical and intuitive decision-making approaches indicates that paramedics rely on a combination of both when managing mental health-related presentations. However, the risks associated with cognitive bias, particularly when intuitive methods are used without sufficient mental health training, highlight the need for better integration of critical thinking frameworks into paramedic practices. Clearer, more flexible guidelines that support decision-making in varied clinical scenarios, rather than rigid procedures, may enhance patient care and reduce errors.

The inconsistent understanding of CPGs and protocols emphasizes the need for more targeted education and training. Educational programs could aim to clarify the distinction between CPGs and protocols, ensuring that paramedics are better prepared to apply critical thinking and adapt their approach based on specific mental health presentations. Additionally, paramedic training might consider incorporating dual process theory to support paramedics in recognizing when to use analytical versus intuitive decision-making. Expanding mental health-specific education may also contribute to reducing stigma and further equip paramedics to manage mental health-related presentations effectively.

This study reveals gaps in how paramedics define clinical decision-making in mental health contexts. Future studies could explore how paramedic education and training influences decision-making and outcomes in mental health-related scenarios. Additionally, further research is needed into the role of CPGs, how paramedics interact with mental health legislation, and the broader influence of experience and organizational culture on clinical decision-making.

A potential limitation to this study is the lack of general consensus on a definition of clinical decision-making in the context of paramedic practice. This was evident from the participant responses, where there was no clear definition of the clinical decision-making process. Another limitation is the variation in scope of practice, CPGs, and policies across different jurisdictions in Australia which may impact the generalizability of the findings.

## Conclusion

This study offers insight into paramedics’ self-perception of clinical decision-making when managing mental health-related presentations. Although not articulated by participants, the dual process theory model incorporating both analytical and intuitive decision-making was evident. Explicitly incorporating dual process theory into education and training, allowing for an understanding of the strengths and weaknesses of both analytical and intuitive decision-making, may potentially reduce clinical errors caused by cognitive bias.

The differing perceptions of CPGs may require a clear distinction between the use of protocols and CPGs to guide critical thinking. Although protocols are useful in chaotic, time-pressured situations, their application may not translate to other presentations that require flexible thinking. To support clinical decision-making, further education and training are warranted to promote critical thinking and clarify scope of practice and role when attending to mental health-related presentations.

## Electronic supplementary material

Below is the link to the electronic supplementary material.

Supplementary Material 1

## Data Availability

The data used to support the findings of this study are available from the corresponding author upon request.
