# Supplementary material for: Paramedic perceptions of decision-making when managing mental health-related presentations: a qualitative study
Source: BMC Med Inform Decis Mak. 2024 Nov 19;24:348. doi: 10.1186/s12911-024-02768-w (PMC11577801; doi:10.1186/s12911-024-02768-w)
Supplement: Supplementary file 1 — Supplementary Material 1 [file 12911_2024_2768_MOESM1_ESM.docx]

**Appendix 1.**

**INTERVIEW SCHEDULE TOPIC GUIDE**

1. **Preamble**

Brief explanation about the study.

We wish to interview paramedics who have had experience managing mental health related presentations.

Provide brief details about the participant’s involvement in study:

- Completion of a short questionnaire; will take approximately 5 minutes to complete.
- Taking part in a tape-recorded interview; will take approximately 45 minutes to complete.

Check the participant has a copy of the Participant Information and Consent Form.

- Give participant time to read the documents.
- Ask if participant has any questions. Answer questions to her/his satisfaction.

Explain the confidential nature of the study and that she/he cannot be identified in the data.

Obtain consent.

Ensure participant gives verbal consent to participate in the study (ensure consent is recorded).

1. **Short questionnaire contains questions about your socio-demographic-occupational circumstances.**

Reinforce that all answers will be treated with complete confidentiality**.**

Write the interview number here:

1. Gender Male 1

Female 2

1. What age are you (to nearest year)?
2. In which State or Territory do you work as a paramedic? (Tick all that apply)

ACT  1

NSW  2

NT  3

Qld  4

SA  5

Vic  6

Tas  7

WA  8

1. In which of the following locations do you work currently as a paramedic? (Tick all that appy

Metropolitan  1

Regional  2

Rural  3

Remote  4

1. What is your current operational role?

Paramedic Manager 1

Paramedic 2

Intensive Care Paramedic 3

Extended Care Paramedic 4

Retrieval Paramedic 5

Flight Paramedic 6

General Care Paramedic 7

Graduate Internship 8

Ambulance Officer 9

First Responder 10

Patient Transport Attendant (Level 1) 11

Patient Transport Attendant (Level2) 12

Basic Life Support Medic 13

Other (please specify) 14

What is your highest level of completed professional education?

Certificate IV 1

Diploma 2

Degree 3

Graduate Certificate 4

Graduate Diploma 5

Masters Degree 6

PhD 7

1. In what year was your highest qualification awarded?
2. Are you an Aboriginal and Torres Strait Islander?

Yes  1

No  2

1. What is your country of birth?

Australia 1

Overseas 2

If born overseas, please state country of birth

1. If born overseas, approximately how long have you lived in Australia?

Years Months N/A

1. How long have you worked as a paramedic in your current place of employment?

Years Months

1. How long have you worked overall as a paramedic?

Years Months

1. **Questions: I am now going to ask you as series of open-ended questions.**

| **INTERVIEW SCHEDULE TOPICS** |
| --- |
| How would you describe your level of knowledge and confidence in managing mental health related presentations? |
| Describe your level of preparedness in managing mental health related presentations |
| In relation to your pre-qualification education how much theory and training was received in mental health?  How adequate was this theory and training to equip you to manage mental health related presentations? |
| What, if any, theory based, and clinical practice “on-road” training have you received about assessing and managing mental health related presentations? |
| What, if any, professional development theory and training is available currently for you about assessing and managing mental health related presentations? |
| What, if any, clinical practice guidelines do your ambulance service have to support your work with mental health related presentations? |
| Aside from education and training, what, if any, access do you have to specialist staff (example: mental health practitioners) to help you manage mental health related presentations? |
| Overall, is there anything else you think of that guides how you manage mental health related presentations? |
